# Obesity during Pregnancy and SARS-CoV-2/COVID-19-Case Series of the Registry Study “COVID-19 Related Obstetric and Neonatal Outcome Study” (CRONOS-Network)

**DOI:** 10.3390/jcm12062089

**Published:** 2023-03-07

**Authors:** Friederike Weschenfelder, Janine Zöllkau, Anna Schohe, Ulrich Pecks, Tanja Groten, Ute Schaefer-Graf, on behalf of CRONOS-Network

**Affiliations:** 1 Department of Obstetrics, University Hospital Jena, 07747 Jena, Germany; 2 Department of Obstetrics and Gynecology, Berlin Center for Diabetes and Pregnancy, St. Joseph Hospital, 12101 Berlin, Germany; 3 Department of Obstetrics and Gynecology, University Hospital of Schleswig-Holstein, 24105 Kiel, Germany

**Keywords:** obesity, overweight, SARS-CoV-2, COVID, complications, pregnancy

## Abstract

(1) Background: Obesity is an increasing challenge in the care of pregnant women. The aim of our study was to investigate whether obesity is an independent risk factor for severe maternal and neonatal outcomes in pregnant women with COVID-19. (2) Methods: Data from the COVID-19 Related Obstetric and Neonatal Outcome Study (CRONOS), a prospective multicenter registry for SARS-CoV-2 positive pregnant women, was used to analyze the effect of obesity on selected individual and combined outcome parameters (3) Results: With 20.1%, the prevalence of obesity in the CRONOS registry exceeds the German background rate of 17.5%. Obese women showed significantly higher rates of GDM (20.4% vs. 7.6%; *p* < 0.001), hypertensive pregnancy disorders (6.2% vs. 2%; *p* = 0.004) and C-sections (50% vs. 34.5%; *p* < 0.001). BMI was revealed to be an individual risk factor for the severe combined pregnancy outcome (maternal death, stillbirth or preterm birth < 32 weeks) (OR 1.050, CI 1.005–1.097). (4) Conclusions: Maternal BMI is a predictor for the most severe outcome as maternal or neonatal death and preterm delivery <32 weeks of gestation. Unexpectedly, categorized obesity seems to have limited independent influence on the course and outcome of pregnancies with COVID infections.

## 1. Introduction

Obesity among reproductive-age women is a common problem with rising numbers. The prevalence of obesity in pregnant women in the 2020 German obstetric inpatient cohort of the Institute for Quality Assurance and Transparency in Health Care was 16.35%, with an increasing trend [1]. In total, 95% of all deliveries in Germany were entered in this national Perinatal database (Institut für Qualitätssicherung und Transparenz im Gesundheitswesen, IQTIG) [1].

Obese women, in general, are at higher risk of pregnancy complications such as hypertensive disorders, preeclampsia, and gestational diabetes mellitus (GDM) and have a higher probability of delivery by C-section.

Compared to non-pregnant individuals, pregnant women with SARS-CoV-2 infection have a higher risk for complications in general and are more frequently admitted to hospitals with a higher risk for ICU admission and need for mechanical ventilation [2,3,4,5]. Preterm delivery rates are higher, and newborns were more likely to be admitted to the neonatal intensive care unit (NICU) [4]. Additionally, several factors seem to increase the risk for severe COVID-19 in pregnancy, such as age, body mass index (BMI) and pre-existing medical disorders. Pregnant women with obesity, pre-existing diabetes or GDM were even more frequently hospitalized for COVID-19-related illness with high mortality and need for ICU admission and mechanical ventilation [6,7,8]. Severe symptoms during SARS-CoV-2 infection and the negative prognosis for obese patients seem to be caused by mechanisms from immune system activity attenuation to chronic inflammation [9].

The aim of this present study was to present data from the “COVID-19 Obstetric and Neonatal Outcome Study” (CRONOS) with a focus on maternal obesity and to compare perinatal and SARS-CoV-2-related outcomes of pregnancies with maternal COVID infection in women considering maternal obesity with these in normal weight women. In addition, the results of our study were compared to the general outcome parameters of the German Perinatal Survey to allow comparability with the general German population.

## 2. Materials and Methods

### 2.1. Study Data Collection

The CRONOS Registry (COVID-19 Related Obstetric and Neonatal Outcome Study) is an ongoing prospective multicenter study for SARS-CoV-2-positive pregnant women, established by the German Society of Perinatal Medicine (DGPM) in April 2020. More information on the study is available at https://www.dgpm-online.org/gesellschaft/forschung/cronos/, accessed on 30 December 2022. Data from the study with a focus on other issues (maternal comorbidities or neonatal aspects) have been recently published [8,10,11,12,13]. Ethical approval was given by the local Ethical Committee (University Hospital Schleswig-Holstein, Kiel, file no. D 451/20). The study was registered in the German Registry for Clinical Studies (DRKS00021208). Cloud-based electronic data capture platform castoredc.com (Amsterdam, The Netherlands) was used for data collection by the participating centers. Data were retrieved from patient records, including basic characteristics, medical history, SARS-CoV-2 infection and treatment, pregnancy and perinatal outcome.

### 2.2. Study Population

**Figure 1:**
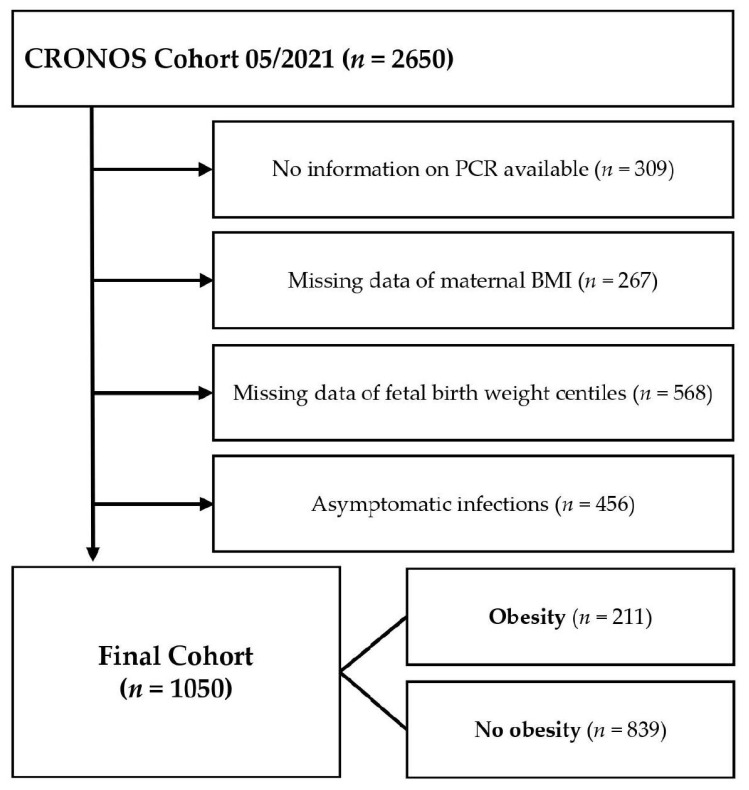
Flowchart of the study cohort. BMI—body mass index; PCR—polymerase chain reaction.

From April 2020 until August 2021, 2650 inpatient pregnant women from 109 centers with diagnosed SARS-CoV-2 infection during pregnancy were registered in the CRONOS registry. We excluded cases without information on PCR results (*n* = 309), cases of missing data on maternal prepregnancy BMI (*n* = 267), pregnancies with missing data of fetal birth weight centiles (*n =* 568) and asymptomatic cases (*n =* 456) from our analysis (See Figure 1). The remaining 1050 datasets were divided into two subgroups depending on maternal prepregnancy BMI, which was calculated from maternal height and the documented prepregnancy weight and further categorized according to the definitions of the World Health Organization [14].

### 2.3. Primary Endpoint Definition

We defined the combined primary endpoints using the defined register variables, as shown in Table 1.

### 2.4. Statistical Analysis

Statistical analysis was performed with SPSS 27.0 (IBM®, New York, NY, USA). The Chi^2^ test or Fisher’s exact test was used to compare categorical data. Most of the continuous data were not normally distributed; therefore, data is presented using the median and interquartile range. Nonparametric tests were used to compare all continuous data between the two subgroups: non-obesity and obesity. Benjamini--Hochberg correction was used for controlling the familywise error rate due to multiple testing [15]. Adjusted odds ratios (aORs) for estimating the association between BMI, maternal age, parity, pre-existing diabetes mellitus, pre-existing cardiovascular diseases, other pre-existing diseases and the combined endpoints (severe combined pregnancy outcome, severe combined neonatal outcome, severe combined maternal outcome, severe combined COVID outcome) were determined using logistic regression. ORs with a 95% confidence interval (CI) are presented. A *p*-value < 0.05 was considered to indicate statistical significance (2-tailed).

## 3. Results

### 3.1. Maternal Parameters

Table 2 shows maternal parameters of all included cases (*n* = 1050): 839 women without obesity and 211 women with obesity (20.1%). Subgroups did not differ concerning maternal age, ethnicity and pre-existing lung diseases. Women in the obesity group were more likely to have pre-existing cardiovascular diseases (7.6% vs. 2.5%, *p* < 0.001), diabetes mellitus (4.3% vs. 0.4%, *p* < 0.001) and other pre-existing diseases (14.2% vs. 5%, *p* < 0.001).

Table A1 shows the BMI categories of the CRONOS cohort of this study compared to the BMI categories of 695.322 women reported in the German obstetric inpatient cohort of the IQTIG [1].

### 3.2. Pregnancy Parameters

Pregnancy and COVID-related parameters are shown in Table 2. Subgroups differed significantly in the rates of GDM (20.4% in the obesity group and 7.6% in the non-obesity-group, *p* < 0.001), occurrence of hypertensive disorders (6.2% vs. 2%, *p* = 0.004) and rates of C-sections (50% vs. 34.5%, *p* = 0.001). There was a higher number of inpatient care treatments in the obesity subgroup (22.7% vs. 16.1%, *p* = 0.041), and more patients with mild diseases (13.8% vs. 11.9%) and severe diseases (8.6% and 4.2%) according to the WHO-OSCI Score (*p* = 0.023). However, both these differences did not remain after using Benjamini--Hochberg correction for multiple testing [15]. We did see a significant difference in the number of obese patients requiring intensive care treatment (8.6% vs. 4.2%, *p* = 0.014). No differences were found for gestational age at diagnosis of COVID-19, symptoms and duration of symptoms.

### 3.3. Neonatal Outcome

Concerning the neonatal outcome, we did not find differences in the number of live births, 5 min APGAR, umbilical cord pH, NICU admission or fetal SARS-CoV-2 diagnostic. We did see significantly more newborns with a birth weight > 4000 g in the obesity group (17.1% vs. 9.3%, *p* < 0.001) and LGA birth weight (18.5% vs. 7.6%; *p* < 0.001) but less SGA newborns (4.3% vs. 7.7% *p* < 0.001). There was a statistical but clinically irrelevant difference in the gestational age at delivery. (See Table 2).

### 3.4. Severe Combined Primary Outcome and Independent Effects

We could not find a significant difference between the two subgroups concerning the combined outcomes (See Table 3). Multivariate analysis revealed maternal age, prepregnancy BMI and parity as independent predictors for the severe combined parameters (See Table 4). For the severe combined pregnancy outcome, only BMI (per BMI-point; aOR 1.050; CI 1.005–1.097) and maternal age (per year aOR 1.076; CI 1.011–1.114) remained significant independent factors. None of the factors was independently associated with severe combined neonatal outcome. Multivariate analysis revealed an independent association of maternal age (per year aOR 1.053; CI 1.009–1.098) and parity (aOR 1.239; CI 1.045–1.468) for the severe combined maternal outcome. Severe combined COVID outcome was only independently affected by maternal parity (aOR 1.310, CI 1.084–1.585).

## 4. Discussion

The aim of this study was to investigate whether obesity is an independent risk factor for perinatal complications in pregnancies affected by SARS-CoV-2 infection that had been documented in the CRONOS register. Only minimal differences are seen regarding the BMI group distribution in our cohort compared to the perinatal data of the IQTIG, suggesting a good representation of the overall German perinatal data within this register (see Appendix A Table A1). With 20.1%, the obesity rate in the CRONOS cohort was marginally higher than the 17.5% in the perinatal statistics [1]. We did see differences in the pregnancy and perinatal outcome data in obese compared to non-obese women with SARS-CoV-2 infections. Obese women showed significantly higher rates of GDM (20.4% vs. 7.6%; *p* < 0.001), hypertensive pregnancy disorders (6.2% vs. 2%; *p* = 0.004) and C-sections (50% vs. 34.5%; *p* < 0.001), as somewhat expected due to the well-known effect of obesity on these outcomes [16,17]. COVID-19 as an indication for C-section was significantly higher in the obesity group (6.7% vs. 2.8%; *p* = 0.011) than in the non-obese group. It remains unclear whether the indication was made out of precaution due to the expected higher morbidity of obese patients or out of acute maternal or fetal indication.

However, no relevant differences in the COVID-related clinical data were found between the two groups besides ICU treatment, which was significantly higher in the obesity group (8.6% vs. 4.2%, *p* = 0.014). Presumably, admission to ICU was more liberally indicated as a precaution since the severe combined maternal outcome was not reported more frequently as in non-obese women. The rate of admitted newborns to NICU in our cohort is higher than the usual rate in the IQTIG database (15.2% vs. 10.8%) [4]. One possible reason for this is the fact that in the early days of COVID-19, children were more often transferred prophylactically to the NICU simply out of precariousness. There was a slightly higher rate of NICU admissions in the obesity group (19% vs. 14.3%, *p* = 0.086), but lacking significance. Interestingly, 12.5% of the admission were only due to their mother’s COVID-19 infection.

Women in the obesity group had more frequently pre-existing medical cardiovascular diseases (7.6% vs. 2.5%; *p* = 0.001), pre-existing diabetes mellitus (4.3% vs. 0.4%; *p* < 0.001) and other pre-existing diseases (14.2% vs. 5%, *p* < 0.001). To eliminate the effect of these pre-existing conditions, a multivariate analysis was performed to evaluate the independent effect of maternal BMI on our primary outcome parameters. BMI was revealed to be a risk factor for maternal death, stillbirth or preterm birth before 32 weeks of gestation (OR 1.050, CI 1.005–1.097). Per BMI unit, the risk increases by 5%, irrespective of other pre-existing diseases. Contrary to our assumptions, BMI was not an individual risk factor for the other primary outcomes: Severe Combined Neonatal Outcome, Severe Combined Maternal Outcome and Severe Combined COVID Outcome.

### 4.1. Context to Existing Data

There are inconsistent data published so far regarding the impact of obesity on the course of SARS-CoV-2 infections in pregnancy. A population-based study included 1332 women with COVID infection delivered within the Kaiser Permanente Hospitals in California and evaluated the risk for perinatal complications and hospital admission. The admission rate was 20%, but obesity was no significant risk factor (OR 1.51; 0.81–2.81); the presence of pregestational diabetes was the only maternal condition that significantly increased the risk for admission [18]. The meta-analysis of Smith et al. with 21,977 cases of SARS-CoV-2 infection in pregnancy or postpartum showed that obesity is a risk factor for severe maternal COVID-19 outcomes, including ICU admission (RR 1.81) or ventilation (RR 2.05) and pneumonia (RR 1.66) [19]. However, similar to our finding, there was no significantly increased risk for fetal or neonatal morbidity or mortality in the presence of obesity besides preterm delivery. The ORs associated with obesity were calculated for each single outcome parameter, in contrast to our approach using combined adverse outcomes.

In the obese subgroup, a higher percentage of mild (13.8% vs. 11.9%) and severe disease (8.6% vs. 4.2%) and a higher need for hospitalization (22.7% vs. 16.1%) was evident, although statistical significance was failed likely due to multiple testing. According to a previous meta-analysis, obesity is an independent risk factor for the severity of symptoms and complications in COVID-19 in the general population, which is consistent with our results [20]. Although our primary outcomes do not show significant differences with respect to severe combined outcomes, a clear trend is seen, especially for severe combined maternal outcomes (12.8% vs. 8.9%) in the obese pregnant group (see Table 3). Pregnant women are already considered a high-risk population for COVID-19, yet pregnant women with higher maternal BMI should be given even higher priority for prevention and treatment.

Just recently, Kleinwechter et al. published their analysis of the CRONOS data with a focus on GDM as a risk factor for adverse maternal and neonatal outcome parameters. Interestingly, GDM itself did not show an independent effect on the defined primary adverse maternal outcome (death, ICU, pneumonia, oxygen supplementation) (OR 1.5, CI 0.88–2.57) nor on the neonatal outcome (stillbirth or neonatal death, NICU). Only in combination with overweight or obese GDM was significantly associated with adverse maternal or neonatal outcome [8]. In contrast to our data, obesity was a significant risk factor for both maternal and neonatal adverse outcomes, although the cohorts overlap, besides the exclusion of cases with missing neonatal birth weight in our cohort. These controversial results demonstrate the influence of the specific selection of parameters to define the combined outcomes used for analysis. A combination of outcome parameters may attenuate or strengthen the association of, e.g., maternal obesity dependent on the selection of the included outcome parameters. However, analysis of data with low prevalence, such as maternal or fetal death, often is not feasible due to low numbers; therefore, the combined outcome is used. Our severe maternal combined outcome (preterm delivery < 32 weeks due to hypertensive pregnancy disorders; PPH > 1500 mL, ICU) consists of parameters partly with different underlying pathophysiology and different severity than those of Kleinwechter; the same is valid for Severe Combined Neonatal Outcome that included SGA, 5min-APGAR < 5 und pH artery < 7.0, preterm delivery < 32 weeks and neonatal death. Smith et al. also showed that pregnant women with chronic comorbidities such as diabetes, cardiovascular disease or hypertension are at higher risk for several outcomes related to COVID-19, including mortality, preeclampsia, hypertensive disorders of pregnancy and C-sections [19]. This leads to the assumption that it is more the combination of obesity and other diseases that lead to an increase in the severity of disease in the context of SARS-CoV-2 infection rather than obesity itself. Therefore, it is even more important that caring physicians are cautious about patients with multiple comorbidities, especially in combination with overweight and obesity. Knowledge about the inflammatory state of obese patients seems to be more relevant and could help in categorizing metabolically healthy and unhealthy obesity, as discussed in the review of Alam et al. Metabolically unhealthy obesity was linked to increased visceral or abdominal fat and more comorbidities. They recommend not focusing on BMI itself but considering the metabolic profile [21].

### 4.2. Strengths and Limitations

One of the strengths of this study data is that the CRONOS registry covers approximately 30% of all deliveries in Germany. Accordingly, the present data set is to be considered representative of the national collective, as also shown in Appendix A Table A1.

As in previous studies, most of our documented cases of pregnant women with SARS-CoV-2 infection were in their late second or third trimester of pregnancy [22]. To exclude incidental findings due to routine screening at hospital admission near term, only symptomatic patients were included in the analysis. Accordingly, we cannot make comparisons between our cohort and women with asymptomatic SARS-CoV-2 infections nor with healthy women. Another general limitation is the problem of categorizing BMI using standardized cut-off values; borderline obese cases with adverse outcomes might have screwed the results. As shown in previous studies of pregnant women with COVID infection [19], underweight women in our cohort experienced high frequencies of perinatal complications that may also have contributed to severe combined outcomes in the non-obese group. Due to lacking information, we could not include social status and quality of maternal care, with known effects on COVID outcome [23].

Another limitation is the aforementioned overlap between the report of Kleinwechter et al. and the present report, which resulted from the simultaneous analyses of the different CRONOS Task Forces with varying focus. The aim of our task force was to investigate the influence of BMI and obesity on pregnancy outcomes. Due to the scientifically proven relevance of BMI on different pregnancy outcomes, it had to be included in the analyses of the other task forces as well. In the end, however, it was shown that BMI has a relevant impact mainly in combination with other diseases [8].

## 5. Conclusions

All our defined outcome categories that combined different maternal and neonatal complications with a wide range of severity are not observed more often in pregnancies of obese than in non-obese women. However, maternal BMI is an independent risk factor for the most serious severe combined pregnancy outcome defined as maternal or neonatal death or preterm birth < 32 SSW but not for the other outcome categories. In our analysis, obesity itself showed a limited influence on the course and outcome of pregnancies with SARS-CoV-2 infections. We assume that potential reasons for these results are the selection of parameters for the definition of the combined outcomes, as well as the influence of borderline overweight women, which possibly bias the results due to the categorization.

## Figures and Tables

**Table 1:** Definition of the Combined Primary Endpoints: Severe combined pregnancy outcome, Severe combined neonatal outcome, Severe combined maternal outcome, Severe combined COVID outcome.

Combined Primary Endpoints	Defining Variables *
Severe combined pregnancy outcome	maternal death neonatal death preterm delivery < 32 weeks
Severe combined neonatal outcome	SGA (<10th Percentile) APGAR 5 min < 5 and pH artery < 7.0 preterm delivery < 32 weeks neonatal death
Severe combined maternal outcome	preterm delivery < 32 weeks due to hypertensive pregnancy disorder postpartum hemorrhage > 1500 mL ICU treatment
Severe combined COVID outcome	NICU treatment maternal ICU treatment invasive maternal ventilation newborn tested positive for SARS-CoV-2 severe COVID-19 disease

ICU—intensive care unit; NICU—neonatal intensive care unit; SGA—small for gestational age; * at least one variable needs to be presented to define a severe combined outcome.

**Table 2:** Cohort characteristics including maternal, pregnancy, COVID-related and neonatal outcome parameters of total cohort (*n* = 1050) and the comparison between women without obesity (*n* = 839) and with obesity (*n* = 211).

Variable	Entire Cohort (*n* = 1050)	No Obesity BMI < 30 kg/m^2^ (*n* = 839; 79.9%)	Obesity BMI > 30 kg/m^2^ (*n* = 211; 20.1%)	*p*-Value
**Maternal Parameters**				
Age (years)	31 (28–34)	31 (28–34)	31 (28–35)	0.807
Parity	1 (0–1)	1 (0–1)	1 (0–2)	0.002 *
BMI (kg/m^2^)	24.5 (21.7–28.7)	23.4 (21.2–26.0)	33.8 (31.6–37.5)	<0.001 *
Caucasian ethnicity	713 (68.4%)	581 (69.7%)	132 (63.5%)	0.052
Cardiovascular disease	37 (3.5%)	21 (2.5%)	16 (7.6%)	0.001 *
Pre-existing diabetes mellitus	11 (1.0%)	2 (0.4%)	9 (4.3%)	<0.001 *
Lung disease	28 (2.7%)	24 (2.9%)	4 (1.9%)	0.307
Other pre-existing diseases	72 (6.9%)	42 (5%)	30 (14.2%)	<0.001 *
**Pregnancy Parameters**				
GDM	107 (10.2%)	64 (7.6%)	43 (20.4%)	<0.001 *
Hypertensive pregnancy disorders	30 (2.9%)	17 (2%)	13 (6.2%)	0.004 *
C-section (any)	394 (37.6%)	289 (34.5%)	105 (50%)	<0.001 *
COVID-19 as indication for C-section	37 (3.5%)	23 (2.8%)	14 (6.7%)	0.011 *
Postpartum hemorrhage (>1500 mL)	44 (4.2%)	36 (4.3%)	8 (3.8%)	0.850
Maternal death	4 (2.2%)	3 (2.3%)	1 (2.2%)	1
**COVID-19-related Parameters**				
GA at diagnosis of COVID-19	31 (24–37)	31 (24–37)	32 (24–37)	0.514
Severe symptoms of COVID-19 (fever, headache or pneumonia)	620 (59%)	496 (59.1%)	124 (58.8%)	0.938
WHO-OSCI				
Very mild disease (Score 1–2)	861 (82.6%)	698 (83.9%)	163 (77.6%)	0.023 †
Mild disease (Score 3–4)	128 (12.3%)	99 (11.9%)	29 (13.8%)	
Severe disease (5–7)	53 (5.1%)	35 (4.2%)	18 (8.6%)	
Inpatient care	181 (17.4%)	134 (16.1%)	47 (22.7%)	0.041 †
Duration of Symptomes				
<7 days	345 (37.8%)	286 (39%)	59 (32.8%)	0.270
7–14 days	394 (43.2%)	308 (42%)	86 (47.8%)	
>14 days	174 (19.1%)	139 (19%)	35 (19.4%)	
Intensive Care Unit	53 (5.1%)	35 (4.2%)	18 (8.6%)	0.014 *
**Neonatal Outcome**				
Birth weight < 1500 g	29 (2.8%)	21 (2.5%)	8 (3.8%)	0.003 *
Birth weight 1500–3999 g	903 (86.3%)	736 (88.1%)	167 (79.1%)	
Birth weight ≥ 4000 g	114 (10.9%)	78 (9.3%)	36 (17.1%)	
SGA	74 (7%)	65 (7.7%)	9 (4.3%)	<0.001 *
LGA	103 (9.8%)	64 (7.6%)	39 (18.5%)	
GA at delivery (weeks)	40 (38–41)	40 (39–41)	40 (38–40)	0.018 *
Preterm birth < 32 weeks	38 (3.6%)	35 (4.2%)	12 (5.6%)	0.095
Live birth	1033 (98.5%)	826 (98.6%)	207 (98.1%)	0.543
APGAR 5 min < 5	19 (1.8%)	13 (1.6%)	6 (2.9%)	0.241
pH artery	7.27 (7.21–7.33)	7.27 (7.21–7.32)	7.29 (7.21–7.34)	0.041 †
pH < 7	5 (0.5%)	5 (0.6%)	0	0.589
NICU admission	159 (15.2%)	119 (14.3%)	40 (19%)	0.086
NICU admission due to mothers’s COVID-19 infection	19 (12.5%)	15 (13.2%)	4 (10.5%)	0.784
**Neonatal SARS-CoV-2 diagnostic**				
Positive SARS-CoV-2 PCR	12 (4.1%)	11 (4.8%)	1 (1.6%)	0.471
Antibodies positive for SARS-CoV-2	175 (64.6%)	138 (64.8%)	37 (63.8%)	0.878

Data are percent or median and interquartile range (IQR) unless otherwise specified. * remaining significant differences (*p* < 0.05) after using Benjamini–Hochberg correction: † not significant (*p* > 0.05) after using Benjamini–Hochberg correction for multiple testing; BMI—body mass index; GA—gestational age; GDM—gestational diabetes mellitus; LGA—large for gestational age; NICU—neonatal intensive care unit; PCR—polymerase chain reaction; PPH—postpartum hemorrhage; SGA—small for gestational age; WHO-OSCI—WHO-Ordinal Scale for Clinical Improvement.

**Table 3:** Severe Combined Primary Outcome for total (*n* = 1050) and the subgroups of women without obesity and with obesity.

Primary Outcome	Entire Cohort (*n* = 1050)	No Obesity BMI < 30 kg/m^2^ (*n* = 839)	Obesity BMI > 30 kg/m^2^ (*n* = 211)	*p*-Value
Severe combined pregnancy outcome	46 (4.4%)	33 (3.9%)	13 (6.2%)	0.186
Severe combined neonatal outcome	103 (9.8%)	87 (10.4%)	16 (7.6%)	0.246
Severe combined maternal outcome	102 (9.7%)	75 (8.9%)	27 (12.8%)	0.092
Severe combined COVID outcome	73 (7%)	53 (6.3%)	20 (9.5%)	0.129

Data are percent significant differences between the two subgroups; severe combined pregnancy outcome (defined as maternal or neonatal death; preterm delivery < 32 weeks of gestation); severe combined neonatal outcome (defined as SGA, 5min-APGAR < 5 und pH artery < 7.0, preterm delivery < 32 weeks of gestation, neonatal death); severe combined maternal outcome (defined as preterm delivery < 32 weeks of gestation due to hypertensive pregnancy disorders; PPH > 1500 mL, Intensive Care Unit); severe combined COVID outcome (defined as neonatal or maternal ICU treatment; invasive maternal ventilation; newborn tested positive for SARS-CoV-2, severe COVID-19 disease); BMI—body mass index; SGA-small for gestational age; ICU—intensive care unit.

**Table 4:** Independent factors associated with the primary outcome parameters: severe combined pregnancy outcome, severe combined neonatal outcome, severe combined maternal outcome, severe combined COVID outcome (*n* = 1037 included cases).

Variable	Severe Combined Pregnancy Outcome	Severe Combined Neonatal Outcome	Severe Combined Maternal Outcome	Severe Combined COVID Outcome
BMI (kg/m^2^)	1.050 * (1.005–1.097)	0.968 (0.929–1.008)	1.026 (0.992–1.061)	1.027 (0.988–1.067)
Maternal age (years)	1.076 * (1.011–1.144)	1.029 (0.988–1.073)	1.053 * (1.009–1.098)	1.005 (0.958–1.055)
Parity	1.021 (0.784–1.330)	0.923 (0.750–1.137)	1.239 * (1.045–1.468)	1.310 * (1.084–1.585)
Pre-existing Diabetes mellitus	2.120 (0.386–11.631)	1.262 (0.149–10.711)	1.509 (0.358–6.359)	1.691 (0.324–8.827)
Pre-existing Cardiovascular disease	1.116 (0.297–4.197)	0.883 (0.259–3.006)	1.070 (0.380–3.010)	0.581 (0.128–2.640)
Other pre-existing diseases	0.734 (0.216–2.496)	0.865 (0.361–2.075)	1.368 (0.665–2.816)	1.303 (0.562–3.022)

* significant independent variables (*p* < 0.05), BMI—body mass index; severe combined pregnancy outcome (defined as maternal or neonatal death; preterm delivery < 32 weeks of gestation); severe combined neonatal outcome (defined as SGA, 5min-APGAR < 5 and pH artery < 7.0, preterm delivery < 32 weeks of gestation, neonatal death); severe combined maternal outcome (defined as preterm delivery < 32 weeks of gestation due to hypertensive pregnancy disorders; PPH > 1500 mL, Intensive Care Unit); severe combined COVID outcome (defined as neonatal or maternal ICU treatment; invasive maternal ventilation; newborn tested positive for SARS-CoV-2, severe COVID-19 disease); SGA-small for gestational age; ICU—intensive care unit. PPH—postpartum hemorrhage.

## Data Availability

The data presented in this study are not publicly available but available on request from the corresponding author. The data are not publicly available due to privacy and ethical restrictions.

## Appendix A

**Table A1:** Comparison of CRONOS cohort data with the German perinatal data (IQTIG).

Variable	IQTIG Cohort 2020 (*n* = 745.804)	Entire Cohort (*n* = 1050)	No Obesity BMI < 30 kg/m^2^ (*n* = 839; 79.9%)	Obesity BMI ≥ 30 kg/m^2^ (*n* = 211; 20.1%)
**BMI categories**	*n* = 695.322			
Underweight	12%	10.8%		
Normal Weight	45.7%	43%		
Overweight	24.7%	26.1%		
Obesity	17.5%	20.1%		
**Maternal Parameters**				
Pre-existing diabetes mellitus	1.3%	1%	0.4%	4.3%
**Pregnancy Parameters**				
GDM	9.5%	10.2%	7.6%	20.4%
Hypertensive pregnancy disorders	2.2%	2.9%	2%	6.2%
C-section (any)	32.2%	37.6%	34.5%	50%
PPH	2.0%	4.2%	4.3%	3.8%
**Neonatal Outcome**				
Birth weight (grams)				
<1500 g	1.4%	2.8%	2.5%	3.8%
1500–3999 g	87.9%	86.3%	88.1%	79.1%
≥4000 g	10.8%	10.9%	9.3%	17.1%
Live birth	99.5%	98.5%	98.6%	98.1%
NICU admission	10.8%	15.2%	14.3%	19%

Data are percent BMI—body mass index; GDM—gestational diabetes mellitus; NICU—neonatal intensive care unit; PPH—postpartum hemorrhage.
